# A Comparison of Sparse Partial Least Squares and Elastic Net in Wavelength Selection on NIR Spectroscopy Data

**DOI:** 10.1155/2019/7314916

**Published:** 2019-08-01

**Authors:** Guang-Hui Fu, Min-Jie Zong, Feng-Hua Wang, Lun-Zhao Yi

**Affiliations:** ^1^School of Science, Kunming University of Science and Technology, Kunming 650500, China; ^2^Faculty of Agriculture and Food, Kunming University of Science and Technology, Kunming, Yunnan 650500, China

## Abstract

Elastic net (Enet) and sparse partial least squares (SPLS) are frequently employed for wavelength selection and model calibration in analysis of near infrared spectroscopy data. Enet and SPLS can perform variable selection and model calibration simultaneously. And they also tend to select wavelength intervals rather than individual wavelengths when the predictors are multicollinear. In this paper, we focus on comparison of Enet and SPLS in interval wavelength selection and model calibration for near infrared spectroscopy data. The results from both simulation and real spectroscopy data show that Enet method tends to select less predictors as key variables than SPLS; thus it gets more parsimony model and brings advantages for model interpretation. SPLS can obtain much lower mean square of prediction error (MSE) than Enet. So SPLS is more suitable when the attention is to get better model fitting accuracy. The above conclusion is still held when coming to performing the strongly correlated NIR spectroscopy data whose predictors present group structures, Enet exhibits more sparse property than SPLS, and the selected predictors (wavelengths) are segmentally successive.

## 1. Introduction

One of characteristics of near infrared spectroscopy (NIR) data is that the number of predictors is much more than the size of observations. Taking corn data [1] as an example, the number of predictors is up to 700 but the sample size is just 80. Thus a problem in building calibration model for NIR is how to select a set of important predictors among a large number of candidate covariates. Wavelength selection for spectroscopy is a classic topic [2] and many methods have been proposed, such as VIP [3], MWPLS [4, 5], and MC-UVE [6]. A drawback of the above algorithms is that model calibration and wavelength selection are separated into two steps: the calibration model is firstly established and then the variable selection procedures are performed based on the model from the first step. Recently, sparse variable selection methods [7–16] have gained much attention for dealing with high-dimensional data from various fields. One of advantages of sparse methods is that they can perform the model calibration and variable selection simultaneously. In addition, sparse algorithm can shrink some estimation coefficients to exactly zero, thus the predictors corresponding to zero-valued coefficients are eliminated from the original calibration model. This is extremely useful when coming to model interpretation. Nowadays, there are many useful sparse methods for addressing the NIR spectroscopy data [17–23]. In this paper, we focus on two of them: elastic net [17] and sparse partial least squares (SPLS) [18]. Both Enet and SPLS can obtain sparse coefficients by choosing appropriate parameters.

Another feature of NIR spectroscopy is multicollinearity among the predictors. The neighboring predictors are continuous wavelength intervals and they are highly correlated. In this situation, the problem is that which strategy should be accepted when doing the model calibration and wavelength selection? In other words, to select a single wavelength each time or an entire interval of strongly correlated and adjacent wavelengths? On one hand, selecting the entire variable group can obtain better calibration and prediction accuracy compared with selecting single predictor from the group when multicollinearity or high correlation is present in the group variables [24–26]. On the other hand, the interval of wavelengths among which the pairwise correlations are strongly correlated should be regarded as a natural group when this wavelength interval is associated with a particular type of chemical bonding. So those predictors in the same group should be in or out of the calibration model simultaneously. For the above two considerations, the sparse methods for NIR spectroscopy data should be able to handle group variables (wavelength intervals) selection, which is called group effect in [17]. Fortunately, both Enet and SPLS can automatically group the multicollinear predictors and select (or eliminate) the entire predictor group simultaneously from the model. Therefore, Enet and SPLS are two potential powerful methods which are suitable for addressing the NIR spectroscopy data. In fact, many references [27–38] have introduced Enet or SPLS to analysis of NIR spectroscopy data. The purpose of this article is to compare the performance of them when dealing with the NIR spectroscopy data.

The remainder of this paper is organized as follows: Section 2 offers the basic theory of Enet and SPLS. Sections 3 and 4 give the experimental results on simulation data and real data sets, respectively. In Section 5, we give the conclusion and make a brief discussion.

## 2. Theory of Enet and SPLS

### 2.1. Sparsity of Enet and SPLS

We consider the following linear model for variable selection and estimation:

(1) $$\begin{array}{c} y=X\beta +\varepsilon =\beta_{1}x_{1}+\beta_{2}x_{2}+\cdots +\beta_{p}x_{p}+\varepsilon , \end{array}$$

where **β** = (*β*_1_, *β*_2_,…,*β*_*p*_)^*T*^ is the regression coefficient vector. **ε** is usually the Gauss noise, namely, **ε** ~ *N*(0, *σ*^2^**I**). **y** = (*y*_1_, *y*_2_,…,*y*_*n*_)^*T*^ is the response and **X** = (**x**_1_, **x**_2_,…, **x**_*p*_) is the predictor matrix, where **x**_*j*_ = (*x*_1*j*_, *x*_2*j*_,…,*x*_*nj*_)^*T*^ is the *j*^*th*^  (*j* = 1,2,…, *p*) predictors. For the simplicity, we also assume that the response variable is centered and the predictors are standardized to have zero mean and unit length, namely,

(2) ∑i=1nyi=0,∑i=1nxij=0,∑i=1nxij2=1,j=1,2,…,p

Traditional methods to obtain the regression coefficients in the linear model (1) are ordinary least squares (OLS). The solution of OLS ${\hat{\beta }}_{\left(OLS\right)}={\left(X^{T}X\right)}^{-1}X^{T}y$ generally has not sparsity (the term “sparsity”, as used here, refers to the linear model (1) having many zero-valued regression coefficients). The OLS is often overfitting and has poor predictive performance when applied to those highly correlated data. To date, there are many ways to deal with this issue. The OLS with the *L*_1_−norm constraint, which is called LASSO [7], may be the most important one [39], as LASSO can perform variable selection and estimation simultaneously.

**Figure 1:**
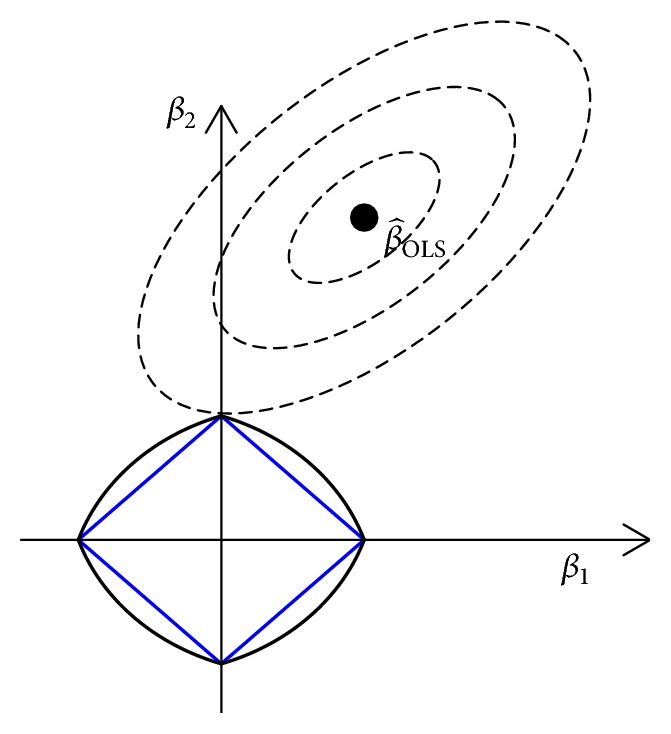
Two-dimensional LASSO penalty (blue) and Enet penalty (black). ${\hat{\beta }}_{\left(OLS\right)}$ is the ordinary least squares solution and the contours reflect the estimates of $\hat{\beta }$ with equal deviation in terms of squared error loss. Enet penalty is strictly convex, so the optimal solution is located in one corner of the Enet.

Enet [17] is an improved version of the LASSO by using doubly regularized parameters and can be expressed by the following constrained OLS optimization problem:

(3) β^Enet=1+λ2·arg⁡minβ⁡y−Xβ22+λ2β22+λ1β1,

where *λ*_1_ and *λ*_2_ are two nonnegative regularization parameters; ‖**β**‖_1_ = ∑_*j*=1_^*p*^|*β*_*j*_| is the *L*_1_-norm; and ‖**β**‖_2_ = (∑_*j*=1_^*p*^*β*_*j*_^2^)^1/2^ is the *L*_2_-norm. If *λ*_2_ = 0, Enet is exactly equivalent to LASSO. The scale factor “1 + *λ*_2_” should be “1 + *λ*_2_/*n*” when the predictors are not standardized to have mean zero and *L*_2_-norm one. Enet penalty “*λ*_2_‖**β**‖_2_^2^ + *λ*_1_∑_*j*=1_^*p*^|*β*_*j*_|” is the combination of *L*_1_-norm and *L*_2_-norm. The *L*_1_-norm constraint induces sparsity; namely, it can shrink those small coefficients being exactly zero. *L*_2_-norm constraint addresses the potential singularity and produces lower prediction error. The Enet constraint can be seen as a mix norm, which is like a fish net (that is why it is called elastic net) (see Figure 1). The Enet ball is a (hyper)cube with corners on the coordinate axes where all but one parameter is exactly zero. It is geometrically easy to see that the loss contours always touches the hypercube in a corner with some of the parameters being exactly zero. So, Enet shrinks some coefficients being exactly zero when the Enet constraint is active.

The important special case comes true when the ridge parameter *λ*_2_ comes to be sufficiently large. In fact, when *λ*_2_ → *∞*, Enet changes to be

(4) $$\begin{array}{c} {\hat{\beta }}_{j^{\left(Enet\right)}}={\left(\;\left|\;y^{T}x_{j}\;\right|-\frac{\lambda_{1}}{2}\;\right)}_{+}\mathrm{sgn}\left(\;y^{T}x_{j}\;\right),\;j=1,2,\ldots ,p, \end{array}$$

where (*z*)_+_ and sgn⁡(*z*) are, respectively, defined as follows:

(5) z+=z,if  z>0,0,if  z≤0.sgn⁡z=1,if  z>0,−1,if  z≤0.

Equation (4) is called univariate soft thresholding (UST) [40] and it shows that Enet coefficients can be estimated by UST when *λ*_2_ is large enough.

Partial least square (PLS) [41–43] is a widely used statistical analytic tool that aims to reduce the dimensionality of the high-dimensional data by constructing latent components. PLS finds the first *K* components by iteration to model the relationship between **X**-matrix and **y**-response. Each component (score) **t** is the linear combination of the original predictors, namely, **t** = **Xw** = *w*_1_**x**_1_ + *w*_2_**x**_2_ + ⋯+*w*_*p*_**x**_*p*_. Generally, each weight *w*_*j*_ of vector **w** obtained by PLS is not zero; thus PLS does not automatically lead to selection of relevant predictors. Although PLS can deal with ill-posed problems and improve the prediction accuracy, it is still hard when coming to model interpretability. So, sparse partial least squares (SPLS) [18] was proposed for getting the sparse solution. Actually, SPLS can be seen as the generalized PLS which inserts a variable selection procedure. SPLS finds its first sparse principal component by the following optimization problem:

(6) minw,c −κwTMw+1−κc−wTMc−w+λ1c1+λ2c22,

(7) s.t. wTw=1,

where **M** = **X**^*T*^**yy**^*T*^**X**, **w**, and **c** are the direction vectors and keep close to each other, 0 < *κ* ≤ 0.5, *λ*_1_ ≥ 0, and *λ*_2_ ≥ 0. Equation (6) can induce the sparse property by imposing the Enet penalty. It should be pointed out that the penalty acts on the surrogate of the direction vector **c** instead of the original direction vector **w**, and **w** and **c** are calculated by an alternative iteration algorithm where solving Enet is a crucial step. For univariate response **y**, $\hat{w}=X^{T}y/\left\|X^{T}y\right\|$ is the direction vector of PLS, and ${\hat{c}}_{j}={\left(\left|{\hat{w}}_{j}\right|-\lambda_{1}/2\right)}_{+}\mathrm{sgn}\left({\hat{w}}_{j}\right)\;\;(j=1,2,\ldots ,p)$ for sufficiently large *λ*_2_. SPLS is also an iteration algorithm that finds first direction vector firstly, then the second and up to figuring out *K* weight vectors.

### 2.2. Group Variables (Wavelength Intervals) Selection of Enet and SPLS

Considering strictly convex of Enet, suppose that *λ*_2_ ≠ 0 and ${\hat{\beta }}_{i^{\left(Enet\right)}}{\hat{\beta }}_{j^{\left(Enet\right)}}>0$ in formula (3), then

(8) $$\begin{array}{c} \left|\;{\hat{\beta }}_{i^{\left(Enet\right)}}-{\hat{\beta }}_{j^{\left(Enet\right)}}\;\right|\leq \frac{\left(1+\lambda_{2}\right){\left\|y\right\|}_{2}}{\lambda_{2}}\sqrt{2\left(1-\rho_{ij}\right)}, \end{array}$$

where *ρ*_*ij*_ = **x**_*i*_^*T*^**x**_*j*_ is the sample correlation coefficient of the predictors **x**_*i*_ and **x**_*j*_. Equation (8) presents an upper bound of the absolute difference of the regression coefficients and indicates that Enet enables group variables (wavelength intervals) selection. Namely, if two predictors are strongly correlated (*ρ*_*ij*_ → 1), the corresponding regression coefficients are almost identical. So those strongly correlated predictors (wavelength intervals) will be simultaneously in or out the model in the form of groups or intervals.

PLS is often calculated by NIPALS [44] and SIMPLS [42] algorithms, but we just employ NIPALS to get SPLS solution in this issue. SPLS- NIPALS can select more than one predictor each time and the response **y** is deflated, so the eigenvector **X**^*T*^**y**/‖**X**^*T*^**y**‖ is proportional to the current correlation. This means that, if there is a group where the predictors are highly correlated, then SPLS can select (or eliminate) these group variables simultaneously.

### 2.3. Tuning the Parameters in Enet and SPLS

Two regularization parameters (*λ*_1_, *λ*_2_) are used in Enet. The sparse parameter *λ*_1_ can be replaced by the fraction (*s*) of the *L*_1_-norm as *s* is limited and ranged from 0 to 1. In practice, *s* can be equally divided into 100 values and the ridge parameter *λ*_2_ can set be some large numbers for the consideration of group effect and UST.

There are totally four parameters (*κ*, *λ*_1_, *λ*_2_, *K*) in the SPLS. A small *κ* (e.g., *κ* = 0.5) is used to avoid local optimization in the iteration. The ridge parameter *λ*_2_ should set to be sufficiently large to obtain a UST solution which just depends on the LASSO penalty parameter *λ*_1_. Thus, just the sparse parameter *λ*_1_ and the number of principal components *K* need to be tuned in practice. In addition, the parameter *λ*_1_ can be replaced by the *η* if the soft thresholding direction vector is set to be

(9) $$\begin{array}{c} \hat{w}=\left(\;\left|\;\hat{w}\;\right|-\eta \;\underset{1\leq j\leq p}{\max}\left|\;{\hat{w}}_{j}\;\right|\;\right)\mathrm{sgn}\left(\;\hat{w}\;\right), \end{array}$$

where 0 ≤ *η* ≤ 1. Compared with *λ*_1_, the advantage of using *η* is that *η* is limited into [0,1]. Thus *η* can be equally divided into 100 intervals in practice. *K* would not be too large; for example, it could be set be 1 to 15. Thus, we make use of 100 × 15 = 1500 grid points to search for the optimal combination of model parameters.

The measurement used for tuning the parameters is mean squared prediction error of tenfold cross-validation (*MSECV*), which is defined as follows:

(10) $$\begin{array}{c} MSECV=\frac{1}{n}\overset{10}{\underset{v=1\;}{\sum }}\underset{i=1}{\sum }{\left(\;y_{i}-{\hat{y}}_{i}^{-v}\;\right)}^{2}, \end{array}$$

where *y*_*i*_ is the measure value of the *i*^*th*^  (*i* = 1,2,…, *n*) sample and ${\hat{y}}_{i}^{-k}$ is the predicted value obtained by leaving the *v*^*th*^ fold samples out.

### 2.4. Computation and Software

The computation and the related procedures are performed with R language [45]. R is a free software environment for statistical computing and graphics [46]. Two packages called “elasticnet” [47] and “spls” [48] are employed respectively in computing Enet and SPLS.

## 3. Simulation Study

The purpose of this section is to give comparisons of Enet and SPLS from several aspects when the true model is known.

### 3.1. Example 1: Study on the Cases of *n* > *p* and *n* < *p*

**Figure 2:**
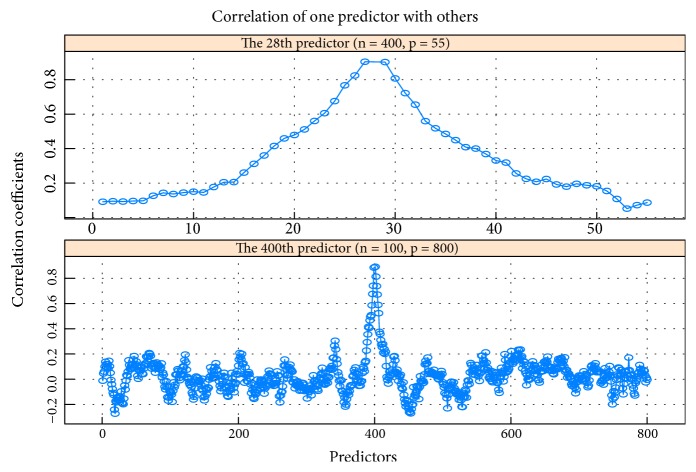
The top subgraph is correlation coefficient path of the 28th predictor with other 54 predictors. The subgraph below is correlation coefficient path of the 400th predictor with other 799 predictors.

In this example, the simulation of overdetermined (*n* > *p*) and underdetermined (*n* < *p*) data sets is used for investing the real-world cases in spectral analysis. We simulate a sparse model with a diverging number of observations, predictors, and sample correlations. The simulation data is generated via the linear model (1) and $\sigma =\sqrt{8}$. The *n* × *p* design matrix **X** is drawn from a multivariate normal distribution *N*(0, Σ) whose covariance matrix Σ has entries Σ_*ij*_ = *ρ*^|*i* − *j*|^, (*i*, *j* = 1,2,…, *p*). Choosing such covariance structure is to coincidence with NIR spectroscopy data as it indicates that those neighboring predictors are more correlated (see Figure 2). We consider *ρ* = 0.5,0.7 and 0.9 and six combinations of (*n*, *p*, *q*): (100, 25, 6), (200, 37, 12), (400, 55, 18), (100, 120, 6), (100, 300, 15), and (100, 800, 35), where *n*, *p*, and *q* are the number of samples, predictors, and nonzero coefficients, respectively, and we suppose that the true coefficients of the first *q* predictors are 3 and the rest are 0, namely,

(11) $$\begin{array}{c} \beta =\left(\;\underset{q}{\underset{︸}{3,3,\ldots ,3}},\underset{p-q}{\underset{︸}{0,0,\ldots ,0}}\;\right) \end{array}$$

Thus 18 combinations of different *n*, *p*, *q*, and *ρ* are discussed, where the first 9 cases are overdetermined and the last 9 cases are underdetermined. The model calibration accuracy is measured by the relative prediction error (*RPE*) defined as follows:

(12) $$\begin{array}{c} RPE=\frac{1}{n\sigma^{2}}\overset{n}{\underset{i=1}{\sum }}{\left(\;{\hat{y}}_{i}-y_{i}\;\right)}^{2}, \end{array}$$

where ${\hat{y}}_{i}$ is the estimate of *y*_*i*_  (*i* = 1,2,…, *n*), and the results for comparisons are listed in Table 1. We can easily see that SPLS outperforms Enet in terms of *RPE* and “C” in almost all the cases, where “C” is the number of predictors that are correctly selected into the model, but SPLS tends to select much more uninformative predictors (denoted by “IC” in Table 1) than Enet. Both Enet and SPLS can select almost all those right predictors contained in the true model and two methods have similar performance in this situation. “C + IC” is the total number of the predictors that are selected into the model, and we can see that Enet tends to select a smaller predictor set as the key variables than SPLS. With the increase of correlation among predictors, the number of predictors selected into the model and the estimation accuracy changes slightly by two methods. In sum, Enet tends to select less predictors as key variables than SPLS; thus it gets more parsimony model and brings advantages for mode interpretation; SPLS can obtain much smaller calibration accuracy than Enet, so SPLS is more suitable when the attention is to get better model fitting accuracy.

### 3.2. Example 2: Comparison of Two Methods That Handle Multicollinearity

**Figure 3:**
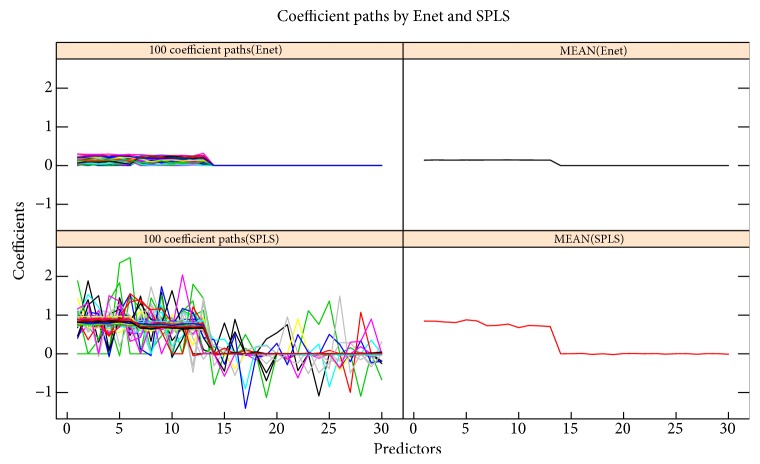
The left subgraphs are the coefficient paths by Enet and SPLS based on 100 replications, and the right subgraphs are the mean of coefficients by the two methods.

It is a good way to perform wavelength intervals selection rather than wavelength points selection in NIR spectroscopy analysis [25]. In this section, we simulate a sparse model to evaluate the group variables selection of Enet and SPLS. We firstly generate three independent latent variables: **v**_*i*_ ~ *N*(0, 5^2^)  (*i* = 1,2, 3), then let the sample size be *n* = 240 and the number of predictors be *p* = 30. The response and 30 predictors are generated as follows:

(13) y=v1+v2+ε,ε~N0,I240xj=v1+εj,if  1≤j≤6,v2+εj,if  7≤j≤13,v3+εj,if  14≤j≤30,

where **ε**_*j*_ ~ *N*(0, **I**_240_)  (*j* = 1,2,…, 30) are independent. We can easily see that the predictors 1 to 6, 7 to 13, and 14 to 30 constitute of three variable group structures, and the predictors in the same group are multicollinear. The first two groups are associated with the response and the third group is mixed into the model as the noise. In this simulation, 100 data sets are generated, and for each data set, the 240 samples are divided into training, validation, and test sets by 120, 60, and 60, respectively. Training set is for building the model, validation set is for tuning model parameters when doing cross-validation, and test set is for testing the performance of the model. Both Enet and SPLS are employed to deal with these 100 data sets, and the corresponding results are shown in Table 2 and Figure 3. We can see that sum up, both Enet, and SPLS have good performance when coming to dealing with strongly correlated data in which the predictors present group structure, this coincides with the theoretical analysis on two methods. Table 2 shows that SPLS performs better than Enet in term with MSE (see (14)). Figure 3 shows that the estimate coefficients of predictors from the same group by Enet are more consistent than that by SPLS. In addition, Enet is more likely to eliminate the uninformative variable groups. We can see that the predictors in the true model (from 1st to 13th predictors) are selected by the Enet and SPLS, but SPLS also select some uninformative predictors (from 14th to 30th predictors) and Enet almost not. So Enet is still the winner when considering variable selection and model interpretation in the case of handling multicollinearity.

## 4. Real Data Sets

Mean square errors (MSE) are utilized as prediction accuracy for real data sets analysis. MSE is defined as follows:

(14) $$\begin{array}{c} MSE=\frac{1}{n}\overset{n}{\underset{i=1}{\sum }}{\left(\;{\hat{y}}_{i}-y_{i}\;\right)}^{2}, \end{array}$$

where ${\hat{y}}_{i}$ is the estimate of *y*_*i*_  (*i* = 1,2,…, *n*) and *n* is the sample size of the data set. In this study, each real data set is divided into training data set and testing data set, and training MSE (Train MSE) and testing MSE (Test MSE) are reported based on 100 replications.

### 4.1. Corn Data Set

**Figure 4:**
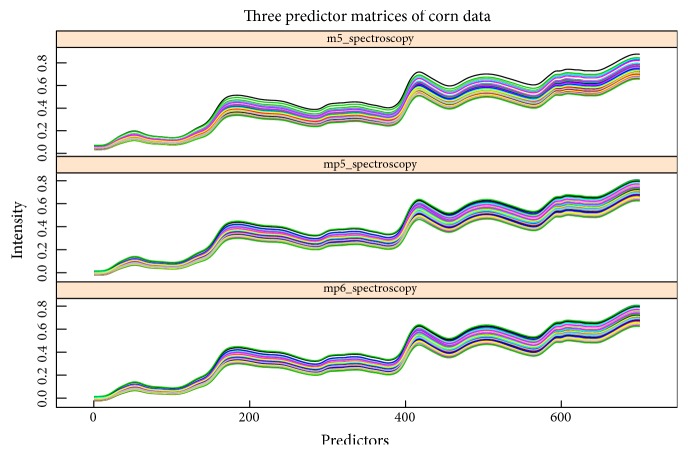
The intensity of each wavelength under three predictor matrices called “m5spec”, “mp5spec”, and “mp6spec” from corn data set. Most of predictors are highly correlated.

The first data set is cited from [1], which consists of 80 samples of corn measured on three different NIR spectrometers. The wavelength range is 1100-2498 nm at 2 nm intervals and thus it gets 700 predictors (or variables) measured by three instruments called “m5”, “mp5”, and “mp6” and correspondingly obtains three predictor matrices called “m5spec”, “mp5spec”, and “mp6spec”, respectively. The predictors of three matrices are generally strongly correlated (see Figure 4). Taking “m5spec” for an example, there are 93.4% predictors whose correlation coefficients are more than 0.92, and even 49.4% predictors whose correlation coefficients are more than 0.99. The moisture, oil, protein, and starch values for each of the samples are also included as response variables and stored in the response matrix “propvals”. In this study, we combine three predictor matrices with four responses to compare the performance of Enet with SPLS.

**Figure 5:**
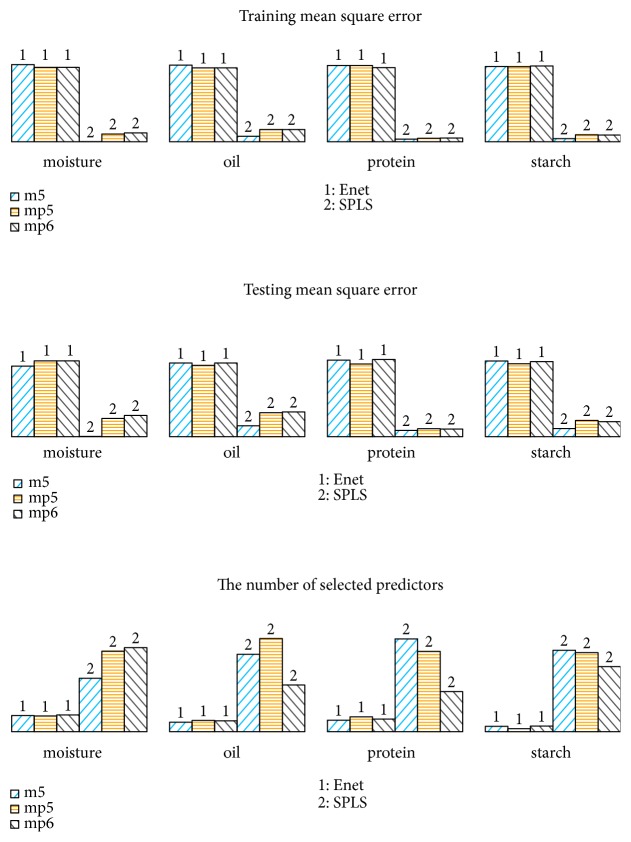
The comparison of Enet and SPLS on corn data set. Three measures “trainMSE”, “testMSE”, and “Num of selected” are scaled to unit one. The results of Enet and SPLS are marked by the numbers “1” and “2”, respectively. The results of three predictor matrices of “m5spec”, “mp5spec”, and “mp6spec” combination of four responses are, respectively, shown by deepskyblue, orange, and grey bars.

**Figure 6:**
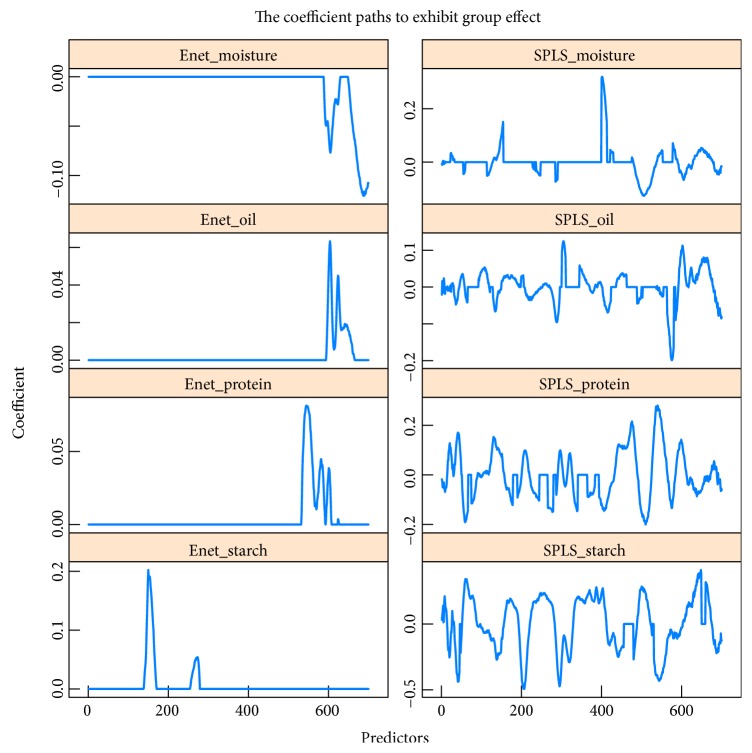
The coefficient paths of predictor matrix “m5spec” with four responses from corn data set. The left and right four panels are generated by Enet and SPLS, respectively. All the panels show that the coefficients paths are segmentally zero-valued or nonzero-valued, so two methods select successive wavelength intervals as key variables.

For each combination, the 80 samples are divided into training set and testing set with the sample size 50 and 30, respectively. The training set is employed to establish the model and the testing set is used to test the model performance. Train MSE, Test MSE, and the number of key predictors (Num of selected) selected into the model are reported based on 100 replications on the data sets. The results are shown in Table 3 and Figures 5 and 6, respectively. Table 3 and Figure 5 tell that SPLS can obtain better calibration accuracy than Enet, but Enet can establish a more sparse model and so it is easier to interpret the model. The above results coincide with the results obtained from simulation data. The testing MSE is close to the training MSE for all the situations by both Enet and SPLS; this illustrates that two methods are suitable for investigating NIR spectroscopy data. Two methods obtained “consistent” results on three predictor matrices with just slight difference, so Enet and SPLS are not sensitive when performing data with noise. In addition, SPLS obtains smaller fitting accuracy but Enet selects much less predictors as key variables. So Enet is more suitable when focusing on model interpretability, and SPLS should be employed when the attention is model calibration accuracy.  Figure 6 tells us that the coefficients paths obtained by two methods are segmentally zero-valued or nonzero-valued. This means that successive wavelength intervals are selected into or eliminated out of the model. Both Enet and SPLS exhibit group effect when performing the NIR spectroscopy data in which the predictors from the neighboring wavelength interval are strongly correlated and can be seen as a group. However, Enet has less variable groups than SPLS, so the group effect is more outstanding by Enet than by SPLS when performing the NIR spectroscopy data.

### 4.2. Gasoline Data Set

**Figure 7:**
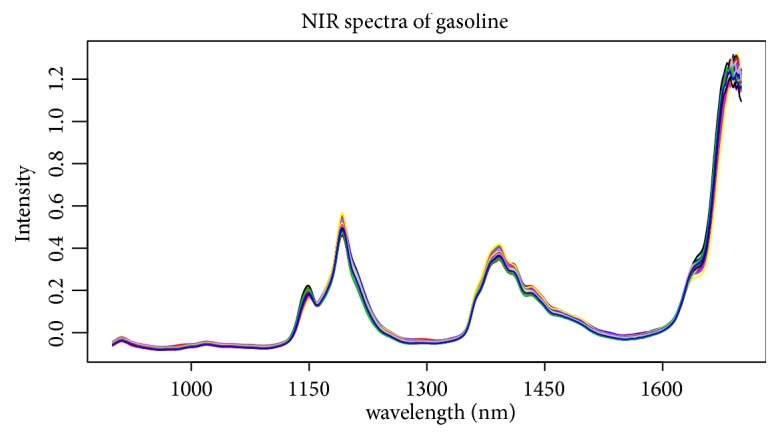
The intensity of each wavelength (predictors) from gasoline data set.

**Figure 8:**
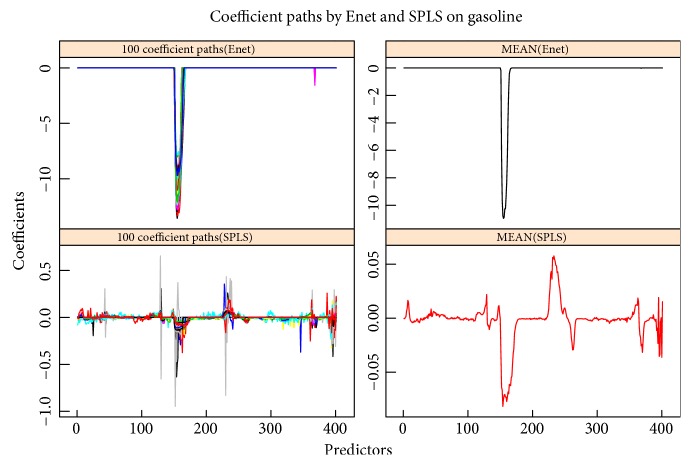
Coefficients paths of gasoline by replicating 100 times. The left subgraphs are the coefficient paths by Enet and SPLS based on 100 replications, and the right subgraphs are the mean of coefficients by the two methods.

The second data set, cited from [49], is another NIR spectral data set with NIR spectra and octane numbers of 60 gasoline samples. The NIR spectra were measured using diffuse reflectance as log(1/R) from 900 nm to 1700 nm in 2 nm intervals, giving 401 wavelengths (predictors) (see Figure 7). 60 samples are also divided into training set and testing set with the sample sizes 38 and 22, respectively. Same as the corn data set, three indices are reported in Table 4 based on 100 replications. Obviously, SPLS has much better estimation accuracy and Enet selects much less predictors as key variables.  Figure 8 shows the regression coefficient paths via 100 replications with randomly choosing the training and testing sets, and it tells that Enet just almost selects one wavelength intervals, but SPLS is not obvious in selecting wavelength intervals, so Enet exhibits much stronger group effect and gets more sparse model than SPLS on gasoline data set.

### 4.3. Buckwheat Data Set

**Figure 9:**
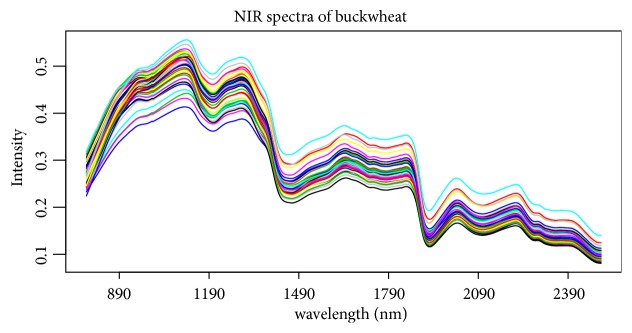
The intensity of each wavelength (predictors) from buckwheat data set.

**Figure 10:**
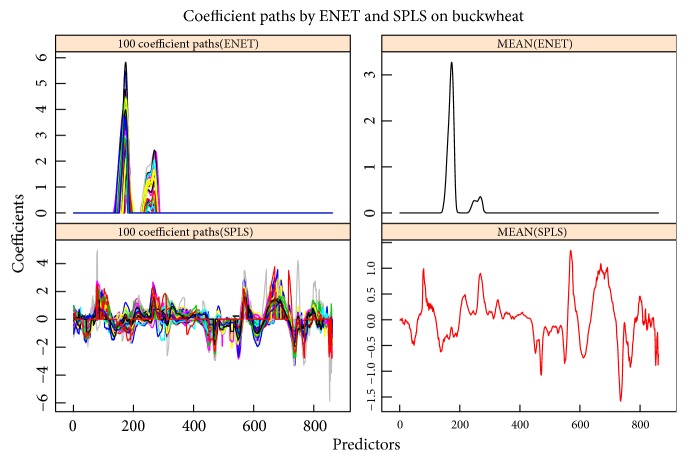
Coefficients paths of buckwheat by replicating 100 times. The left subgraphs are the coefficient paths by Enet and SPLS based on 100 replications, and the right subgraphs are the mean of coefficients by the two methods.

The above corn and gasoline are two public NIR spectroscopy data sets, and the third NIR spectroscopy data set, called “bwX”, is from our lab, which consists of 40 observations of buckwheat measured by FieldSpec 3 spectrometer. The NIR spectroscopy wavelength range is 780-2500 nm at 2 nm intervals; thus it contains 861 predictors. The NIR spectra were measured using diffuse reflectance as log⁡(1/R) (see Figure 9). Starch in buckwheat is measured as the response in this study (called “bwy”). The starch is the vital nutrient in buckwheat and the fast detection of starch is very important in practice. 40 samples are also divided into training set and testing set with the sample sizes 30 and 10, respectively. 100 replications are performed on the buckwheat data sets and the results are reported in Table 5 and Figure 10. Similar to the results from gasoline data set, Table 5 and Figure 10 still show that SPLS obtains much low prediction error and Enet is more likely to select less wavelength intervals or predictors as important variables.

## 5. Conclusion and Discussion

Enet and SPLS are two popular model calibration and selection methods for dealing with NIR spectroscopy data. The number of predictors of NIR data is much larger than sample size and the neighboring predictors are continuous, multicollinear wavelength intervals. The two methods can not only select more predictors than sample size but also exhibit group effect. In other words, Enet and SPLS can automatically group the multicollinear predictors and select or eliminate the entire predictor group simultaneously from the model for the “large p and small n” data. So the two methods are very suitable for investigating NIR spectroscopy data. The purpose of this article is to try to give advice on which method should be used when dealing with NIR data in practice. The results from both simulation and real spectroscopy data show that Enet tends to select less predictors as key variables than SPLS; thus it gets more parsimony and sparse model and brings advantages for mode interpretation. SPLS can obtain much smaller model calibration accuracy than Enet. So SPLS is more suitable when the attention is to get better fitting accuracy. What is more important, the above conclusion is still held when coming to performing the strongly correlated data whose predictors present group structures. In addition, two methods can obtain “consistent” results when the predictor matrices present slight differences, so they are not sensitive when performing data with noises.

As mentioned above, SPLS tends to select a large number of predictors when performing the high-dimensional NIR spectroscopy data. Although the reference of SPLS [18] states that (6) is proposed to obtain a sufficiently sparse solution, it is not so sparse in practice, especially compared with Enet. In this situation, one can also use two or more steps to further shrink the size of predictors. In other words, one can firstly employ SPLS to roughly select the predictors and then use other sparse methods such as Enet to refine the rest candidate predictors.

## Figures and Tables

**Table 1:** Comparison of Enet and SPLS under combinations of different *n*, *p*, *q* and *ρ* based on the 100 replications. *RPE* is the relative prediction error, “C” and “IC” are the number of predictors that are correctly and incorrectly selected into the model, respectively.

		*ρ* = 0.5			*ρ* = 0.7			*ρ* = 0.9		
*n*/*p*/*q*	Model	*RPE*	C	IC	*RPE*	C	IC	*RPE*	C	IC
100/25/6	Truth		6.000	0.000		6.000	0.000		6.000	0.000
	Enet	0.737	5.930	0.160	0.590	5.940	0.130	0.128	6.000	1.150
	SPLS	0.180	6.000	1.810	0.140	6.000	1.630	0.172	5.950	2.610

200/37/12	Truth		12.000	0.000		12.000	0.000		12.000	0.000
	Enet	2.796	11.440	0.020	5.500	10.320	0.000	7.937	9.820	0.000
	SPLS	0.121	11.990	1.440	0.140	11.990	1.900	0.230	11.990	6.080

400/55/18	Truth		18.000	0.000		18.000	0.000		18.000	0.000
	Enet	4.516	17.290	0.030	12.770	14.780	0.000	16.153	12.920	0.000
	SPLS	0.097	18.000	2.020	0.110	18.000	2.830	0.204	17.980	6.710

100/120/6	Truth		6.000	0.000		6.000	0.000		6.000	0.000
	Enet	0.709	5.930	0.200	5.530	6.000	0.700	5.555	6.000	1.970
	SPLS	0.124	5.990	0.480	0.190	5.990	0.760	0.246	5.970	2.990

100/300/15	Truth		15.000	0.000		15.000	0.000		15.000	0.000
	Enet	5.837	13.550	2.420	10.640	12.090	0.020	8.191	12.130	0.010
	SPLS	0.434	14.930	4.460	0.370	14.960	2.820	0.679	14.850	4.660

100/800/35	Truth		35.000	0.000		35.000	0.000		35.000	0.000
	Enet	33.360	25.300	18.000	50.732	22.670	0.860	6511.400	32.480	0.890
	SPLS	2.440	28.000	33.670	1.620	32.600	17.200	1.730	34.590	7.030

**Table 2:** Model selection and fitting results based on 100 replications in studying of multicollinearity. “MEAN” and “SD” denote mean and standard deviation, respectively.

	*MSE*		*C*		*IC*	
*Model*	MEAN	SD	MEAN	SD	MEAN	SD
Enet	34.928	0.432	12.860	0.569	0.000	0.000

SPLS	1.343	0.012	12.730	1.016	1.610	3.315

**Table 3:** The results on “corn” data set based on 100 replications.

Method	**X**	**y**	Train MSECV(SD)	Test MSE(SD)	Num of selected
Enet	m5spec	moisture	0.083(0.010)	0.082(0.019)	95.120(22.881)
		oil	0.028(0.004)	0.031(0.008)	68.420(24.923)
		protein	0.213(0.024)	0.239(0.043)	75.870(24.947)
		starch	0.645(0.061)	0.703(0.131)	43.120(29.281)
SPLS	m5spec	moisture	0.000(0.000)	0.000(0.000)	316.010(31.807)
		oil	0.002(0.000)	0.005(0.001)	561.370(100.538)
		protein	0.007(0.001)	0.020(0.006)	612.560(76.857)
		starch	0.027(0.005)	0.076(0.024)	657.480(51.408)
Enet	mp5spec	moisture	0.080(0.010)	0.088(0.016)	92.720(19.693)
		oil	0.027(0.003)	0.030(0.006)	81.820(21.233)
		protein	0.213(0.022)	0.227(0.040)	98.230(17.267)
		starch	0.644(0.070)	0.678(0.140)	23.830(20.286)
SPLS	mp5spec	moisture	0.008(0.001)	0.021(0.047)	477.020(47.843)
		oil	0.005(0.001)	0.010(0.003)	675.730(38.556)
		protein	0.010(0.002)	0.025(0.008)	530.010(63.240)
		starch	0.060(0.010)	0.151(0.052)	637.700(45.997)
Enet	mp6spec	moisture	0.080(0.011)	0.088(0.020)	99.180(24.223)
		oil	0.027(0.003)	0.031(0.006)	78.620(19.547)
		protein	0.207(0.021)	0.241(0.038)	82.990(29.517)
		starch	0.650(0.070)	0.698(0.117)	44.720(30.092)
SPLS	mp6spec	moisture	0.010(0.002)	0.025(0.007)	497.520(50.199)
		oil	0.005(0.001)	0.010(0.003)	338.410(73.079)
		protein	0.010(0.002)	0.024(0.008)	264.360(56.396)
		starch	0.058(0.010)	0.140(0.041)	525.090(101.531)

**Table 4:** The results on “gasoline” data set based on 100 replications.

	Train MSE(SD)	Test MSE(SD)	Num of selected(SD)
Enet	0.741(0.113)	0.869(0.487)	12.590(1.710)

SPLS	0.026(0.009)	0.060(0.021)	167.650(86.003)

**Table 5:** The results on “buckwheat” data set based on 100 replications.

	Train MSECV(SD)	Test MSE(SD)	Num of selected(SD)
Enet	12.743(1.429)	15.359(4.862)	48.500(17.020)

SPLS	2.696(1.576)	10.354(5.492)	658.580(142.789)

## Data Availability

Three real data sets used in the following section as well as corresponding instructions are available in the electronic supplementary material (available here). The corn [1] as well as gasoline [49] data sets is two public spectroscopy data sets, and the buckwheat data set is from our lab and can be used freely.

## Supplementary Materials

Supplementary Materials Three real data sets used in Section 4. All the data sets are saved as “.txt” format and can be used freely.
